# The psychometric properties of the grazing questionnaire in an obesity sample with and without binge eating disorder

**DOI:** 10.1186/s40337-022-00604-y

**Published:** 2022-06-16

**Authors:** Dean Spirou, Jayanthi Raman, Mimi Leith, James Collison, Ramy H. Bishay, Golo Ahlenstiel, Phillipa Hay, Evelyn Smith

**Affiliations:** 1 Discipline of Clinical Psychology, Graduate School of Health, University of Technology Sydney, Sydney, NSW Australia; 2 Blacktown Metabolic and Weight Loss Program, Department of Endocrinology and Diabetes, Blacktown Hospital, Blacktown, Sydney, NSW Australia; 3 School of Medicine, Western Sydney University, Sydney, NSW Australia; 4 School of Psychological Sciences, University of Newcastle, Callaghan, NSW Australia; 5 School of Psychology, Western Sydney University, Sydney, NSW Australia; 6 Australian College of Applied Professions, Sydney, NSW Australia; 7 Storr Liver Centre, Westmead Millennium Institute, Westmead Hospital, University of Sydney, Sydney, NSW Australia; 8 Department of Gastroenterology and Hepatology, Blacktown and Mount Druitt Hospitals, WSLHD, Sydney, NSW Australia; 9 Camden and Campbelltown Hospitals, SWSLHD, Campbelltown, Australia; 10 Translational Health Research Institute, Western Sydney University, Sydney, NSW Australia

**Keywords:** Obesity, Grazing, Binge eating disorder, Eating disorders, Grazing questionnaire, Psychometric properties, Factor structure

## Abstract

**Background:**

Despite being the first validated measure of grazing, the Grazing Questionnaire (GQ) has not been investigated among individuals with obesity. Therefore, the current study aimed to examine the psychometric properties of the GQ in an obesity sample.

**Methods:**

Participants (*N* = 259) were recruited from community and clinical settings in Australia. The sample comprised adults with normal weight (*n* = 77) and obesity (*n* = 182). A portion of individuals with obesity (*n* = 102) had binge eating disorder (BED). Data from the obesity group was examined to establish the factor structure, validity, and reliability of the GQ. A one-way ANOVA with planned contrasts was conducted to compare scores on the GQ across groups.

**Results:**

Confirmatory factor analysis revealed that the 2-factor model of the GQ was the best model fit for individuals with obesity. The GQ demonstrated high internal consistency, test–retest reliability over 3 months, and convergent and divergent validity. As hypothesised, the obesity group had significantly higher scores on the GQ than the normal weight group, while the obesity with BED group had significantly higher scores than the obesity without BED group.

**Conclusion:**

This was the first study to investigate the psychometric properties of the GQ in an obesity sample. Overall, findings indicated that the GQ is a psychometrically sound measure of grazing among individuals with obesity. These findings provide further support for two distinct subtypes of grazing and highlight the importance of increased assessment and management of grazing behaviours for individuals with obesity and eating disorders.

**Plain English summary:**

Maintaining a healthy weight is one of the greatest challenges for individuals with obesity. Certain eating patterns such as grazing may contribute to difficulties in weight management. Grazing is the repetitive and unplanned eating of small amounts of food that is not related to feeling hungry. Researchers and clinicians often use self-report questionnaires to measure grazing. However, the first validated questionnaire of grazing has not been investigated among individuals with obesity. Therefore, the goal of this study was to examine and validate the Grazing Questionnaire in individuals with obesity. Overall, our results showed that the Grazing Questionnaire is a valid and reliable self-report measure of grazing in individuals with obesity. Similar to previous research, we found that there are two subtypes of grazing. The first subtype involves continuous, unplanned eating. The second subtype is associated with a sense of loss of control over eating. We also found that people with obesity and binge eating disorder graze more than people with obesity that do not have binge eating disorder, while both groups graze more than individuals with normal weight. We recommend that clinicians routinely assess and treat unhelpful grazing patterns when working with individuals with obesity and eating disorders.

## Introduction

Long-term weight management involves the successful navigation of diet and physical activity and remains the greatest obstacle for individuals with obesity [1]. Problematic and disordered eating behaviours complicate weight management and may be a hindrance to treatment outcomes. Research on problematic and disordered eating in obesity has predominantly focused on binge eating, with limited attention on other eating patterns [2]. More recently, however, research has begun investigating the role of grazing behaviour among individuals with obesity and eating disorders.

A recent expert consensus defined grazing as an eating behaviour characterised by the repetitive and unplanned consumption of small amounts of food that is not associated with the sensation of hunger [3]. Research has found that grazing is highly prevalent in obesity and eating disorders, especially binge eating disorder (BED) [4–6]. Binge eating is characterised by eating an objectively large amount of food in a discrete period accompanied by a sense of loss of control over eating [7]. Individuals with BED have consistently shown a higher frequency of grazing compared to those without BED across community [8] and obesity treatment samples [6, 9]. Previous research has suggested that binge eating may be a self-regulatory response to emotion dysregulation [10], but it remains unclear whether grazing serves a similar function and warrants further investigation. Interestingly, researchers have suggested that pre-bariatric surgery binge eating may develop into post-surgery grazing due to the anatomical restrictions of surgery [11, 12]. However, some researchers contend that there is insufficient evidence for this claim [4]. Similarly, there are inconsistent findings on the relationship between pre-treatment grazing and obesity treatment outcomes as well the relationship between grazing and psychological distress, binge eating, and quality of life [4]. On the other hand, research has demonstrated that post-bariatric surgery grazing is often associated with adverse treatment outcomes, including less weight loss, weight regain, and gastrointestinal complications [4].

Three validated self-report measures have been developed to specifically assess the grazing construct, including the Grazing Questionnaire (GQ) [2], the Repetitive Eating Questionnaire, Rep(eat)-Q [13], and the Short Inventory of Grazing (SIG) [14]. The 7-item GQ has been validated in normal weight Australian and Italian university students [2, 15]. The initial development study of the GQ found a 2-factor model of grazing, comprising (1) continuous eating behaviours and (2) perceived loss of control over eating [2]. The GQ was later translated into Italian with the 2-factor structure showing good fit via confirmatory factor analysis in normal weight Italian university students [15]. Furthermore, the Rep(eat)-Q has been validated in normal weight Norwegian and Portuguese populations [13, 16] and in a Portuguese bariatric population [13], with a 2-factor model of grazing evident across all studies. Finally, the 2-item SIG assesses grazing in general and compulsive grazing, and has been validated in a normal weight university and community Australian sample [14].

To date, the factor structure and psychometric properties of the GQ have not been examined among individuals with obesity, despite being the first validated measure of grazing. Validating the GQ in individuals with obesity will extend previous findings [2, 15] and could improve our ability to comment on the utility of this measure in relation to other validated measures of grazing. It will also contribute to coalescing both the theoretical and empirical considerations of this measure. In addition, the inclusion of a BED subgroup will assist with clarifying previous findings [6, 8, 9] that were reported using non-validated scales, such as the differences in grazing between individuals with and without BED.

The primary aim of the current study was to examine the psychometric properties and 2-factor structure of the GQ in a sample of individuals with obesity. To our knowledge, this is the first study to investigate the psychometric properties and factor structure of the GQ in an obesity sample. In addition, we aimed to examine whether grazing varied among individuals with obesity with and without BED and normal weight controls. To our knowledge, this is the first study to include a BED subgroup and to compare grazing patterns across groups using a validated measure. Further, were examined the relationship between grazing and emotion dysregulation, a factor implicated in weight management that has received less clinical attention.

In light of previous literature, we hypothesised that the GQ would demonstrate good model fit as well as high internal consistency and test–retest reliability. We also hypothesised that the GQ would demonstrate convergent validity as evidenced by significant and positive associations with binge eating and eating psychopathology. Similarly, we predicted that the GQ would show divergent validity, as illustrated by a weak and non-significant association with physical quality of life. Further, we hypothesised that grazing would be significantly and positively associated with emotion dysregulation. Moreover, we predicted that grazing would be significantly higher in the obesity group compared to the normal weight control group. We also predicted that grazing would be significantly higher in the obesity with BED group compared to the obesity without BED group.

## Method

### Participants

Participants were 291 adults from Australia, recruited via advertisements in community/university noticeboards, social media, and from the Blacktown Metabolic and Weight Loss Program at Blacktown Hospital. The sample comprised individuals with obesity (*n* = 186) and normal weight controls (*n* = 105). A portion of individuals with obesity also had BED (*n* = 102), as assessed by a trained clinician in accordance with the Diagnostic and Statistical Manual of Mental Disorders-fifth edition (DSM-5) criteria [7]. Recruitment took place over a 5-year period. All participants were notified about the research aims prior to consenting. Participants were eligible if body mass index (BMI) was between 18.5 and 24.9 kg/m^2^ (normal weight) or $$\ge$$ 30 kg/m^2^ (obesity). Participants were excluded if they had a history of psychosis, head injury, neurological disorder, degenerative or inflammatory conditions, stroke, epilepsy, substance dependence, and developmental or intellectual disability. Participants were also excluded if they had a hearing or vision impediment, cognitive impairment, or mental health condition that precluded the completion of testing, or if they were regularly using stimulant medication, hypnotics, antipsychotics, or cholinergic medications. Eligible participants completed a series of self-report questionnaires via research electronic data capture (REDCap), a secure web-based software platform [17, 18]. A $15 gift voucher was offered to community participants as reimbursement. The study was approved by the Western Sydney Local Health District (5450 – 2019/ETH01915) and was ratified by the University of Technology Sydney (ETH20-5199; ETH20-5545).

Of the 291 participants, 32 were excluded due to incomplete data. The final sample (*N* = 259) comprised males (27.8%) and females (72.2%) that ranged in age from 18 to 79 years (*M* = 41.16, *SD* = 11.94) and ranged in BMI from 18.5 to 75.4 kg/m^2^ (*M* = 37.38, *SD* = 13.25). Of these 259 participants, 77 (29.7%) were in the normal weight control group, 80 (30.9%) were in the obesity without BED group, and 102 (39.4%) were in the obesity with BED group. Table 1 presents demographic characteristics of the final sample.

**Table 1:** Demographic characteristics of each group and the overall sample

Variable	NWC (*n* = 77)	O-NonBED (*n* = 80)	O-BED (*n* = 102)	Total (*N* = 259)
Age^a^	34.56 (11.14)	44.90 (12.56)	43.22 (9.95)	41.16 (11.94)
BMI^a^	22.15 (1.99)	45.22 (10.96)	47.73 (9.84)	37.38 (13.25)
Sex^b^				
Male	25 (32.5%)	23 (28.7%)	24 (23.5%)	72 (27.8%)
Female	52 (67.5%)	57 (71.3%)	78 (76.5%)	182 (72.2%)
Highest education level^b^				
Less than Year 10	–	6 (7.5%)	7 (6.9%)	13 (5.0%)
High school (Year 10)	2 (2.6%)	18 (22.5%)	12 (11.8%)	32 (12.4%)
High school (Year 12)	13 (16.9%)	11 (13.8%)	9 (8.8%)	33 (12.7%)
College/TAFE ^b^	6 (7.7%)	24 (30.0%)	30 (29.4%)	60 (23.2%)
Bachelor’s degree	23 (29.9%)	8 (10.0%)	25 (24.5%)	56 (21.6%)
Master’s degree	30 (39.0%)	13 (16.3%)	19 (18.6%)	62 (23.9%)
Doctoral degree	3 (3.9%)	–	–	3 (1.2%)
Employment status^b^				
Employed	52 (67.5%)	45 (56.3%)	75 (73.5%)	172 (66.4%)
Unemployed	5 (6.5%)	28 (35.0%)	21 (20.6%)	54 (20.8%)
Studying	20 (26.0%)	1 (1.3%)	2 (2.0%)	23 (8.9%)
Retired	–	6 (7.5%)	4 (3.9%)	10 (3.9%)

Missing values not included. *BMI* body mass index, *NWC* normal weight control, *O-BED* obesity with binge eating disorder, *O-NonBED* obesity without binge eating disorder

^a^*M* (*SD)*. ^b^*n* (*%*)

### Measures

#### Demographics and eligibility screening

A general self-report questionnaire collected sociodemographic information including age, sex, highest education level, and employment status. Physical and mental health screening items were also included in the questionnaire to assist with determining participant eligibility.

#### Anthropometric measures

Participant weight and height was measured using standardised scales. Self-reported weight and height was used for a portion of the normal weight group (*n* = 30) due to procedural variations associated with COVID-19. BMI was calculated by dividing weight by height (kg/m^2^).

#### BED diagnosis

BED diagnosis was assessed by a trained clinician (e.g., clinical psychologist) via semi-structured interviews in accordance with the DSM-5 criteria [7].

#### Grazing questionnaire (GQ)

The GQ is a 7-item self-report measure of food grazing behaviours [2]. The GQ measures the unplanned, continuous eating of small amounts of food and the loss of control over the amount of food eaten. The GQ comprises two subscales: Grazing Behaviours (items 1–4) and Controllability (items 5–7). Items are rated on a 5-point Likert scale ranging from 0 (*never*) to 4 (*all of the time*). A total score ranging from 0 to 28 is derived by summing each individual item. Higher scores indicate greater grazing cognitions and behaviours. The GQ has been validated in an Australian and Italian university sample and has demonstrated high internal consistency among the total score (Cronbach’s α = 0.82) [2, 15], Grazing Behaviours subscale (Cronbach’s α = 0.83) [15], and Controllability subscale (Cronbach’s α = 0.77) [15]. The GQ has also shown adequate (intraclass correlation = 0.62–0.71) [2] to high (intraclass correlation = 0.88–0.92) [15] test–retest reliability.

#### Eating disorder examination questionnaire (EDE-Q)

The EDE-Q is a 28-item self-report measure of eating disorder psychopathology [19]. The EDE-Q comprises a global score and four subscale scores: Weight Concern, Eating Concern, Shape Concern, and Restraint. Higher scores indicate greater eating disorder psychopathology. The EDE-Q also provides frequency data on key behavioural features of eating disorders. In this study, item 14 was used to measure binge eating frequency. The psychometric properties of the EDE-Q have been extensively examined and its reliability and validity are supported [20]. In the current study, the internal consistency of the global score and subscales ranged from 0.70–0.86.

#### Difficulties in emotion regulation scale (DERS)

The DERS is an 18-item self-report measure of emotion regulation [21]. The DERS yields a total score and six subscale scores: Difficulties Engaging in Goal-Directed Behaviour, Non-Acceptance of Emotional Responses, Impulse Control Difficulties, Lack of Emotional Clarity, Lack of Emotional Awareness, and Limited Access to Emotion Regulation Strategies. Higher scores indicate greater emotion regulation difficulties. The DERS has demonstrated high internal consistency and good concurrent, convergent, and predictive validity [21]. In the current study, the internal consistency of the total score and subscales ranged from 0.81–0.92.

#### 12-Item short-form health survey (SF-12)

The SF-12 is a 12-item self-report measure of health-related quality of life [22]. The SF-12 generates two summary scales: Mental Component Summary (MCS) and Physical Component Summary (PCS). These scores provide a measure of mental health-related quality of life and physical health-related quality of life, respectively. Higher scores indicate better mental and physical functioning. In this study, divergent validity was assessed by examining the correlation between grazing and the PCS. This approach was also utilised by Heriseanu et al. [14] in their validation of the SIG. The SF-12 has demonstrated good psychometric properties in clinical and non-clinical populations across various countries [23–26]. In the current study, the internal consistency of the PCS scale was 0.78.

### Data analyses

Initially, the data was cleaned, and assumption testing was completed. Listwise deletion was used to manage missing data. To test the 2-factor structure of the GQ, confirmatory factor analysis (CFA) was performed using the maximum likelihood estimation method. CFA is the preferred method to evaluate a latent model when there is existing theory on the structure of the data [27]. The root-mean-square error of approximation (RMSEA), standardised root-mean-square residual (SRMR), Tucker-Lewis index (TLI), comparative fit index (CFI), and model chi-square statistic (χ^2^) were examined to determine model fit. A non-significant (*p* > 0.05) χ^2^ indicates good fit [28, 29]. RMSEA values < 0.08 indicate acceptable fit while values < 0.05 indicate good fit [28, 29]. SRMR values < 0.08, TLI values $\ge$ 0.95, and CFI values $\ge$ 0.90 indicate good fit [28, 29]. To examine the stability of the GQ across groups, the factorial invariance of the GQ was tested. The internal consistency of the GQ was assessed using Cronbach’s α. Cronbach’s α values above 0.7 are generally considered acceptable for psychological constructs [30]. To evaluate the test stability of the GQ, a test–retest reliability analysis was performed by comparing scores on the GQ at baseline and 3 months later on a subsample of individuals with obesity. To examine the convergent validity of the GQ, Pearson correlations were conducted between the GQ and similar disordered eating constructs, including the EDE-Q binge eating frequency score and global score. To examine the divergent validity of the GQ, Pearson correlations were conducted between the GQ and the PCS of the SF-12. In addition, a one-way analysis of variance (ANOVA) with planned orthogonal contrasts was conducted to examine whether groups differed on the GQ. Specifically, the normal weight control group was compared to the obesity group (contrast 1), while the obesity without BED group was compared to the obesity with BED group (contrast 2). CFA was performed in SPSS Amos Version 27.0, while all other analyses were carried out in SPSS Version 27.0.

## Results

### Data screening

Data from the sample of participants with obesity (*n* = 182) was examined to establish the factor structure, validity, and reliability of the GQ in this population. Although the Kolmogorov–Smirnov test indicated a non-normal distribution *D*(182) = 0.076, *p* = 0.012, examination of the P–P and Q–Q plots and the values of skew and kurtosis indicated a normal distribution. In addition, the assumption of independence was met due to the study design, and there were no significant outliers.

### Confirmatory factor analysis

**Fig. 1:**
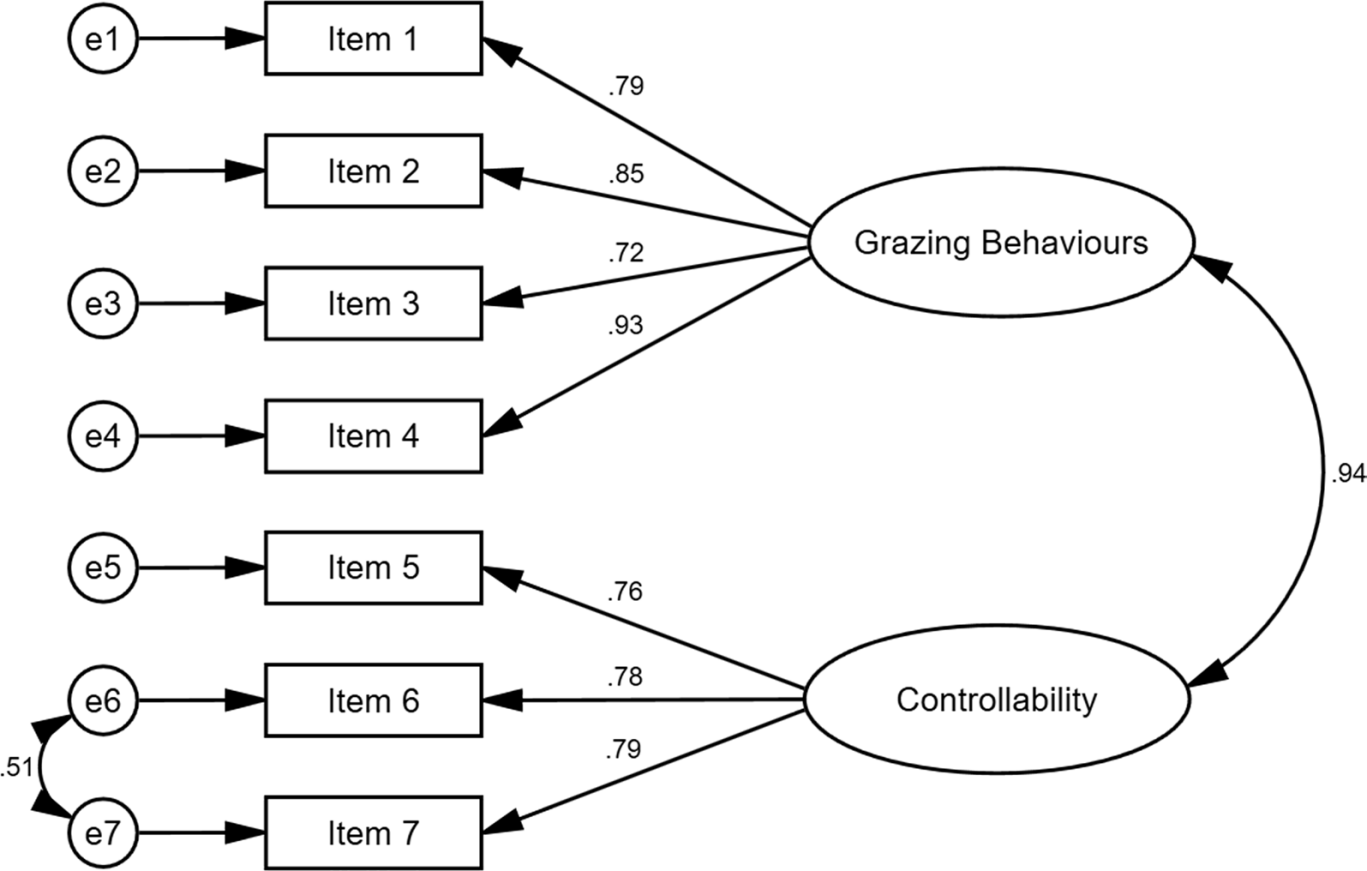
The best-fitting model of the grazing questionnaire in obesity

The hypothesised 2-factor model (Model 1), χ^2^ (13, *N* = 182) = 34.88, *p* = 0.001, RMSEA = 0.096, CFI = 0.98, TLI = 0.96, SRMR = 0.04, produced χ^2^ and RMSEA values that indicated a marginal fit. A respecified 2-factor model (Model 2) was considered with correlated residuals introduced for item 6 (*have you ever felt that you were unable to stop “grazing”?*) and item 7 (*do you have a feeling that you have lost control over your eating while “grazing”?*). These correlated residuals were added to the model due to the conceptual overlap in item content and phrasing, with both items measuring the perceived ability to stop or control grazing. Model 2, χ^2^ (12, *N* = 182) = 16.23, *p* = 0.181, RMSEA = 0.044, CFI = 1.00, TLI = 0.99, SRMR = 0.02, produced χ^2^, RMSEA, CFI, TLI, and SRMR values that indicated a good fit. Further, Model 2 produced a statistically better fit than Model 1, Δχ^2^ = 18.65, *df* = 1, *p* < 0.001. A 1-factor model (Model 3) of the GQ was also compared to the respecified 2-factor model (Model 2). Although Model 3, χ^2^ (13, *N* = 182) = 20.59, *p* = 0.081, RMSEA = 0.057, CFI = 0.99, TLI = 0.99, SRMR = 0.03, demonstrated an acceptable to good fit, Model 2 produced a statistically better fit than Model 3, Δχ^2^ = 4.36, *df* = 1, *p* < 0.05. Overall, Model 2 (Fig. 1) demonstrated a statistically better fit than Model 1 and Model 3 and was the best-fitting model of the GQ among individuals with obesity. Table 2 presents the goodness-of-fit statistics for all the models.

**Table 2:** Goodness-of-fit statistics for the confirmatory factor analysis models

Model	χ^2^	*df*	*p*	RMSEA	CFI	TLI	SRMR	Δχ^2^	Δ*df*
Model 1^a^	38.44	13	0.001	0.096	0.98	0.96	0.04	–	–
Model 2^b^	16.23	12	0.181	0.044	1.00	0.99	0.02	–	–
Model 3^c^	20.59	13	0.081	0.057	0.99	0.99	0.03	–	–
Model 1 versus Model 2	–	–	–	–	–	–	–	18.65**	1
Model 3 versus Model 2	–	–	–	–	–	–	–	4.36*	1

*CFI* comparative fit index, *RMSEA* root-mean-square error of approximation, *SRMR* standardised-root-mean square residual, *TLI* Tucker–Lewis index

^a^Hypothesised 2-factor model. ^b^Respecified 2-factor model. ^c^1-factor model

*p < 0.05. **p < 0.001

The factorial invariance of the GQ was also tested across the obesity with BED group, obesity without BED group, and normal weight control group. A comparison of the unconstrained and fully constrained model revealed no significant difference, Δχ^2^ = 26.62, *df* = 18, *p* > 0.05, suggesting that all factor loadings, variances, covariances, and one error covariance, were invariant across the three groups.

### Validity and reliability

#### Internal consistency

As hypothesised, the Grazing Behaviours subscale (Cronbach’s α = 0.89), Controllability subscale (Cronbach’s α = 0.86), and total score (Cronbach’s α = 0.92) demonstrated high internal consistency.

#### Test–retest reliability

As predicted, the GQ demonstrated high test–retest reliability among 26 participants with obesity, 3 months after initial administration (Cronbach’s α = 0.86, mean intraclass correlation = 0.83).

#### Convergent and divergent validity

Consistent with our hypothesis, the GQ demonstrated good convergent validity, as evidenced by significant and positive correlations with eating disorder psychopathology (*r* = 0.26, *p* = 0.001) and binge eating frequency (*r* = 0.39, *p* < 0.001). Similarly, consistent with our prediction, the GQ showed good divergent validity, as illustrated by a weak and non-significant association with the PCS of the SF-12 (*r* = 0.15, *p* = 0.059).

Further analyses were conducted to examine the relationship between grazing and emotion dysregulation (DERS), a factor that has been shown to directly affect binge eating and weight management [31]. As hypothesised, grazing was significantly and positively correlated with emotion dysregulation (*r* = 0.51, *p* < 0.001). Table 3 presents the intercorrelations and Table 4 presents the descriptive statistics for these measures.

**Table 3:** Intercorrelations among the measures for the obesity group

Variable	1	2	3	4	5
1. GQ	–				
2. EDE-Q global	0.26*	–			
3. Binge eating	0.39**	0.25*	–		
4. DERS	0.51**	0.36**	0.27**	–	
5. SF-12 PCS	0.15	0.012	− 0.094	0.086	–

*DERS* difficulties in emotion regulation scale, *EDE-Q* eating disorder examination questionnaire, *GQ* grazing questionnaire, *SF-12 PCS* 12-item short-form healthy survey physical component summary

*p < 0.05. **p < 0.001

**Table 4:** Means and standard deviations of the measures

Variable	NWC (*n* = 77)	O-NonBED (*n* = 80)	O-BED (*n* = 102)	Total (*N* = 259)
GQ	8.66 (4.96)	11.66 (5.82)	17.52 (5.43)	13.08 (6.59)
EDE-Q global	0.79 (0.91)	2.66 (1.09)	3.22 (.89)	2.32 (1.40)
Binge eating	0.39 (0.89)	0.60 (1.00)	12.52 (8.82)	5.23 (8.10)
DERS	36.96 (11.63)	40.73 (12.72)	48.90 (13.25)	42.83 (13.58)
SF-12 PCS	53.14 (5.29)	34.18 (10.30)	35.23 (8.01)	38.36 (11.14)

*DERS* difficulties in emotion regulation scale, *EDE-Q* eating disorder examination questionnaire, *GQ* grazing questionnaire, *NWC* normal weight control, *O-BED* obesity with binge eating disorder, *O-NonBED* obesity without binge eating disorder, *SF-12 PCS* 12-item short-form healthy survey physical component summary

### Comparing the GQ across groups

To compare scores on the GQ across groups, a one-way ANOVA with planned contrasts was conducted utilising the total sample (*N* = 259). Initial examination of the data revealed one significant outlier, which was excluded from analyses. The assumption of normality, linearity, and independence were met, and Levene’s test indicated equal variances among groups *F*(2, 256) = 1.233, *p* = 0.293. A one-way ANOVA revealed a significant effect of group on grazing, *F*(2, 256) = 62.51, *p* < 0.001, ω = 0.32. As predicted, planned contrasts revealed that individuals with obesity had significantly higher scores on the GQ compared to normal weight controls *t*(256) = 8.03, *p* < 0.001, *r* = 0.45. As hypothesised, of the participants with obesity, individuals with BED had significantly higher scores on the GQ compared to individuals without BED *t*(256) = 7.24, *p* < 0.001, *r* = 0.41.

## Discussion

The current study was the first to examine the factor structure, validity, and reliability of the 7-item GQ in a sample of individuals with obesity. In addition, this was the first study to use a validated measure of grazing to compare patterns across individuals with obesity with and without BED and normal weight controls.

The findings of this study indicated that the 2-factor model of the GQ was the best model fit for individuals with obesity. Importantly, this 2-factor model was invariant across groups, highlighting the stability of the factor structure. These results are consistent with previous research on the GQ that found a 2-factor model in normal weight university students [2, 15]. They are also consistent with previous literature on the Rep(eat)-Q that found a 2-factor model in normal weight and bariatric populations [13, 16]. Together, these findings contribute to the growing consensus that there are two patterns or subtypes of grazing that emerge across different weight classes.

The notion of different grazing patterns or subtypes has been put forth by previous researchers. Conceição et al. [3] proposed the idea of a compulsive and non-compulsive subtype of grazing. The compulsive subtype is characterised by an inability to resist eating and incorporates a sense of loss of control, while the non-compulsive subtype is characterised by eating in a mindless or distracted manner over a prolonged period [3]. Studies utilising the Rep(eat)-Q and SIG have supported the proposition of two grazing subtypes and have suggested that compulsive grazing exists on a disordered eating continuum [13, 14, 16]. These grazing subtypes are conceptually similar to the Grazing Behaviours (non-compulsive grazing) and Controllability (compulsive grazing) subscales of the GQ. Interestingly, Lane and Szabó [2] contemplated whether a 1-factor model would emerge in obesity, indicating that individuals with obesity engage in a grazing pattern predominantly characterised by loss of control. Our results contributed to elucidating this question by demonstrating that the 2-factor model of the GQ produced a better fit than the 1-factor model. This finding reinforces the notion that grazing is better characterised by two subtypes across individuals with normal weight and obesity.

As expected, the GQ demonstrated high internal consistency and test–retest reliability across a 3-month interval. These findings extend previous studies [2, 15] and support the reliability of the GQ in an obesity sample. In addition, consistent with our hypothesis and previous research [5, 14, 16, 32], the GQ demonstrated good convergent validity, as evidenced by significant and positive correlations with eating disorder psychopathology and binge eating. Similarly, consistent with our prediction, the GQ demonstrated good divergent validity, as illustrated by a weak and non-significant correlation with the PCS of the SF-12. Furthermore, we examined the association between grazing and emotion dysregulation, a factor implicated in weight management [31]. As predicted, grazing was significantly and positively correlated with emotion dysregulation. Indeed, this correlation was larger than the one found with eating disorder psychopathology and binge eating. Given the relationship between grazing and both emotion dysregulation and binge eating, it is plausible that grazing may function to alleviate emotion dysregulation, similar to binge eating. Before this conclusion can be made, however, research must clarify whether grazing is an independent disordered eating behaviour or forms part of another eating disorder such as BED.

As hypothesised, individuals with obesity had significantly higher scores on the GQ compared to normal weight controls. This result is consistent with previous research that found a significantly higher BMI among those with compulsive grazing relative to those with non-compulsive or no grazing [5, 14]. Similarly, although limited by a small sample size, Heriseanu et al. [14] found that participants with severe loss of control grazing were the only subgroup in their study with an average BMI in the obesity range. Taken together, these findings support the notion that individuals with obesity exhibit a higher frequency of grazing compared to normal weight populations. Furthermore, consistent with our hypothesis and previous research [6, 8, 9], individuals with BED had significantly higher scores on the GQ compared to those without BED. This finding extends previous studies by using a validated measure of grazing to assess group differences. Although previous research has found that individuals with BED are more likely to have obesity [33], it will be important for future studies to replicate these findings in normal weight populations with BED.

### Implications

The findings of this study contribute to the growing literature on grazing patterns among individuals with normal weight and obesity. Consistent with previous research [2, 3, 5, 14], we found that most individuals engage in some level of grazing, suggesting that it is a common eating behaviour in the population. However, the frequency of grazing may significantly vary across subgroups. For instance, our findings indicated that grazing scores were highest among individuals with obesity and BED and lowest among normal weight individuals. Furthermore, previous research has indicated that the type of grazing pattern may also significantly vary, with certain patterns associated with greater clinical impairment. For example, relative to non-compulsive grazing, grazing with the presence of loss of control is associated with more eating disorder symptoms, more severe eating disorder psychopathology, higher BMI, greater psychological distress, and lower mental health-related quality of life [5, 14, 16, 32]. Consistent with previous literature [13], this suggests that it is the loss of control component of grazing that is associated with psychological distress and eating psychopathology rather than grazing itself. This supports the view that grazing exists on a continuum, with compulsive grazing more likely to reflect a problematic or disordered eating presentation.

Furthermore, although evidence suggests that grazing is a ubiquitous eating behaviour in the population [2, 3, 5, 14], problematic grazing patterns may occur more frequently among individuals with obesity, especially bariatric patients. For instance, post-surgery grazing may represent a unique disordered eating behaviour that develops due to the anatomical restrictions of surgery [11, 12]. The function of this eating pattern, however, may differ among patients. For example, post-surgery grazing may reflect efforts to binge eating or regulate emotion, or may be a result of physiological adaptations to eating. Irrespective of the function, though, post-surgery grazing has been shown to hinder weight loss outcomes and contribute to gastrointestinal complications [4]. Post-surgery grazing may also contribute to psychological distress, especially if characterised by a sense of loss of control. Therefore, grazing, in addition to other disordered eating behaviours, should be routinely assessed after bariatric surgery through a multi-method approach (e.g., self-report questionnaire, clinical interview). This will aid clinicians in identifying the eating patterns that emerge post-surgery as well as their function, and could contribute to improved post-surgical outcomes.

### Limitations and future directions

In light of the current findings, there are several limitations and key areas that could be explored to progress the field. First, our findings were interpreted within the broader grazing literature. Although results were consistent with previous research, they may not be comparable due to nuances in the operationalisation of grazing [14]. Previous researchers have noted the differences and similarities of the existing grazing measures, including their relative utility and limitations (see [14]). As this was the first study to find a 2-factor model of the GQ in obesity, future research is required to verify the validity of these results. Furthermore, future research could continue investigating other validated measures of grazing. For example, the SIG could be examined among clinical groups (e.g., individuals with obesity and eating disorders), while the Rep(eat)-Q could be validated in an Australian normal weight and obesity sample.

Second, although the sex distribution in our study was comparable to previous research on the GQ [2, 15], previous studies predominantly recruited younger, normal weight university students, while we recruited older, community and treatment-seeking individuals with obesity. Future research could replicate these findings across various age groups, which may improve the external validity of these results. Similarly, future research could examine the test–retest reliability of the GQ over a shorter period (e.g., 3 weeks), which would be more ideal for examining the stability of this construct across time.

Third, as all our constructs were examined using self-report methods, treatment-seeking individuals with obesity may have underreported the severity of their eating disorder symptoms due to concerns of ineligibility for treatment [34]. In addition, a portion of normal weight participants self-reported their weight and height due to procedural variations associated with COVID-19. Although these participants were vulnerable to socially desirable responding, previous research has asserted that the limitations of self-report methods are often overstated [35]. Further, the use of semi-structured interview to assess BED circumvents potential self-report bias, and is a strength of this study.

Fourth, the model fit significantly improved when correlated residuals were introduced for item 6 and item 7. Both these items measure the feeling that one has been unable to stop grazing or has lost control over grazing. In other words, both items measure the individual’s perceived sense of loss of control over grazing. Given the theoretical overlap in item content and phrasing, future research could consider refining the wording of these items to enhance the operationalisation of the grazing construct. Further, as loss of control is a defining element of several eating behaviours (e.g., grazing, binge eating), future reviews could explore loss of control as a transdiagnostic feature of eating disorders.

## Conclusion

This was the first study to investigate the psychometric properties of the GQ in an obesity sample. Overall, findings indicated that the GQ is a psychometrically sound self-report measure of grazing among individuals with obesity. Results revealed that the 2-factor model of the GQ was the best model fit for individuals with obesity. These results provide further support for two distinct subtypes of grazing, namely continuous unplanned eating (non-compulsive grazing) and a sense of loss of control (compulsive grazing). In addition, this study was the first to compare grazing patterns across groups using a validated measure. Results indicated that grazing frequency varied across groups, with grazing scores highest among individuals with obesity and BED. Given the potential impact of grazing on eating psychopathology, psychological distress, and treatment outcomes, grazing should be routinely assessed prior to and following weight interventions, especially bariatric surgery. Increased assessment and management of grazing could contribute to improving longer-term outcomes for individuals with obesity and eating disorders.

## Data Availability

The datasets used and/or analysed during the current study are available from the corresponding author on reasonable request.
